# The risk of rifampicin-resistant TB after drug-susceptible TB treatment

**DOI:** 10.5588/ijtldopen.24.0552

**Published:** 2025-03-12

**Authors:** A.N. Shapiro, H. Moultrie, K.R. Jacobson, J. Bor, P. da Silva, L. Scott, H.E. Jenkins

**Affiliations:** ^1^ Department of Biostatistics, Boston University School of Public Health, Boston, MA, USA;; ^2^ Health Economics and Epidemiology Research Office, Department of Internal Medicine, School of Clinical Medicine, Faculty of Health Sciences, University of the Witwatersrand, Johannesburg, South Africa;; ^3^ Centre for Tuberculosis, National Institute for Communicable Diseases, a division of the National Health Laboratory Services, Johannesburg, South Africa;; ^4^ Division of Infectious Diseases, Boston Medical Center, Boston, MA, USA;; ^5^ Department of Global Health, Boston University School of Public Health, Boston, MA, USA;; ^6^ National Priority Program, National Health Laboratory Service, Johannesburg, South Africa;; ^7^ Wits Diagnostic Innovation Hub, Faculty of Health Sciences, University of the Witwatersrand, Johannesburg, South Africa.

**Keywords:** tuberculosis, recurrent TB, drug-resistant TB, rifampicin-resistant TB

Dear Editor,

Recurrent TB contributes substantially to the total disease burden, particularly in high-burden countries such as South Africa.^1^ Recurrent TB is caused by either continued infection from the same strain that caused the initial TB episode (relapse) or infection from a new strain after successful treatment (reinfection). Treatment for drug-susceptible (DS) TB is a risk factor for drug-resistant TB (DR-TB) development.^2^ We therefore assessed a cohort of people with confirmed rifampicin-susceptible TB (RS-TB) to quantify their rifampicin-resistant TB (RR-TB) risk.

The study was based on data from South Africa’s public health care system between January 1, 2013, and October 30, 2015, 2 years following the initial RS-TB episode start date. Because the dataset lacks treatment outcomes, we defined an initial episode to start on the person’s first positive TB test date and end on the latter of either 6 months after the initial positive RS-TB test, or the last negative culture or smear test, provided the individual has a negative smear or culture test followed by at least 6 months without another positive test. An individual was eligible for a recurrence 6 months after the prior episode ended. Due to GeneXpert’s (Cepheid, Sunnyvale, CA, USA) lower specificity among those with previously diagnosed TB,^3^ a positive smear or culture result was necessary to confirm recurrence. We stratified the risk by sex, age, HIV status, local background RR-TB notification rates, and isoniazid resistance (INHR) in the initial episode. We also examined the time between the initial RS-TB episode and RR-TB recurrence. The cohort and definitions are further described elsewhere.^4^

Of the 360,787 people with a confirmed RS-TB episode eligible for a recurrence, 15,134 (4.2%) people had a recurrence and 1,043 (0.29%) of those had RR-TB at recurrence. Males had 1.3 (95% confidence interval [CI] 1.1–1.5) times the RR-TB risk upon recurrence compared to females. People living with HIV (PLHIV) had 2.6 (95% CI 2.5–2.8) times the risk compared to people not living with HIV (PnLHIV). These trends were similar among all recurrences regardless of drug resistance.^4^ The RR-TB risk at recurrence was highest among people aged 25–34 years (0.4%) compared to other age groups and the RR-TB risk decreased with decreasing background RR-TB notification rates (Table); 78% of RR-TB recurrences occurred within 6 months of a person becoming eligible for a recurrence (6 months after the presumed end of their first episode). Median time until recurrence following an RS-TB initial episode was 3.1 months (interquartile range [IQR] 1.2–5.9) for those with an RR-TB recurrence and 4.2 months (IQR 1.7–8.1) for those with an RS-TB recurrence; these values were significantly different using the Wilcoxon rank sum test (*P* < 0.001). Only 387 (37%) of those with an RR-TB recurrence and 3,991 (28%) of those with an RS-TB recurrence received INHR testing during their initial episode. Among people who received INHR testing and subsequently had a recurrence, those with INHR-TB in their initial episode had 3.6 (95% CI 3.0–4.4) times the RR-TB recurrence risk, compared to those who did not have INHR-TB in their initial episode. Males and PLHIV with INHR-TB in their initial episode had equal risk (1.1 times, 95% CI 0.8–1.4; 1.02 times, 95% CI 0.7–1.5, respectively) of having RR-TB in their recurrent episode compared to PnLHIV and females. The RR-TB recurrence risk following an initial INHR-TB episode increased with increasing background RR-TB notification rates but was not associated with age. We could not differentiate between RR-TB recurrences that were relapses or reinfection with a new RR-TB strain, due to the lack of treatment outcomes and/or genomic profiles. However, the high concentration of recurrences occurring soon after the initial infection is suggestive of a higher proportion of relapses, although they could also be due to treatment interruptions or loss to follow-up.^5^ A modelling study revealed that undiagnosed DR-TB was the leading cause of DR-TB amongst recurrence cases.^6^ Additionally, some RR mutations are not captured on GeneXpert, a primary tool in the diagnostic pathway.^7^ We found that people with RR-TB recurrence had a statistically significantly shorter time to recurrence than those with RS-TB recurrence, possibly due to undiagnosed RR-TB at baseline, as other studies suggest,^6,7^ but possibly also due to resistance development during treatment. A prospective cohort study in South Africa from the same period examined acquired RR-TB rates among people with an initial RS-TB diagnosis and found a 2.9% relapse rate, with one-third (0.97%) of these people acquiring RR-TB.^8^ Our cohort includes only individuals who sought care, had a positive symptom screen, and produced a bacteriologically negative sputum sample after treatment to confirm recurrence eligibility. Furthermore, there may have been additional individuals not counted in our cohort who died or were lost to follow-up. Therefore, our results may be under-estimates.

**Table.** RR-TB recurrences among those eligible for a TB recurrence.*

	Patients with initial confirmed RS-TB episode and RR-TB recurrence *n*	Patients eligible for recurrence *n*	Risk of recurrent RR-TB among those eligible for recurrence %	Risk ratio (95% CI)
Total	1,043	360,787	0.29	
Sex				
Female	383	151,726	0.25	Reference
Male	660	207,427	0.32	1.28 (1.13–1.45)
Age, years				
<15	11	7,672	0.14	0.53 (0.06–4.96)
15–24	140	52,102	0.27	Reference
25–34	414	109,240	0.38	1.41 (1.16–1.71)
35–44	314	93,736	0.33	1.25 (1.02–1.53)
45–54	126	53,712	0.23	0.87 (0.68–1.11)
55–64	38	24,503	0.16	0.58 (0.41–0.83)
≥65	0			
HIV status				
Negative	254	164,984	0.15	Reference
Positive	789	195,803	0.40	2.62 (2.48–2.76)
RR-TB notification rates				
Q1 (lowest)	173	98,756	0.18	
Q2	256	87,477	0.29	1.67 (1.48–1.86)
Q3	168	60,070	0.28	1.60 (1.39–1.81)
Q4 (highest)	405	92,889	0.44	2.49 (2.31–2.67)
Missing	41	21,595	0.19	1.08 (0.74–1.42)
Province				
Eastern Cape	222	67,325	0.33	Reference
Free State	56	22,262	0.25	0.76 (0.47–1.05)
Gauteng	119	45,886	0.26	0.79 (0.57–1.01)
KwaZulu-Natal	170	70,542	0.24	0.73 (0.53–0.93)
Limpopo	42	18,520	0.23	0.69 (0.36–1.02)
Mpumalanga	50	18,542	0.27	0.82 (0.51–1.13)
Northern Cape	55	12,372	0.44	1.35 (1.06–1.64)
North West	80	23,249	0.34	1.04 (0.78–1.3)
Western Cape	208	60,495	0.34	1.04 (0.85–1.23)
Missing	41	21,594	0.19	0.58 (0.25–0.91)

* RR-TB notification rates are calculated as the number of people with notified RR-TB in a municipality divided by the total population of the municipality.

RR-TB = rifampicin-resistant TB; RS-TB = rifampicin-susceptible TB.

Of particular concern is the increased RR-TB risk among those who had a recurrence and had previously been tested and were positive for INHR-TB. This finding echoes the results of a meta-analysis, which found that INHR-TB is a risk factor for RR-TB development.^9^ They found that 3.5% of patients with INHR-TB developed multidrug-resistant TB (MDR-TB; i.e., acquired rifampicin resistance in addition to the already present INHR) during treatment compared with 0.2% of participants with INH-susceptible TB. Less than 30% of our cohort had INHR testing and hence were likely put on continuation treatment phase where they would have been exposed to rifampicin as a sole effective agent. These low INHR testing rates are seen in other low- and middle-income countries and are attributed to limited laboratory capacity and increased GeneXpert use, which tests only for RR-TB.^10,11^ A modeling study of PnLHIV found that most acquired MDR-TB was among patients with INHR-TB.^12^ In conjunction with our results, this suggests increased INHR testing and less reliance on GeneXpert alone for treatment decisions are critical to prevent further MDR-TB development and spread. In 2024, South Africa implemented two additional WHO-endorsed moderate complexity nucleic acid amplification tests: the Roche Cobas^®^ MTB (Roche, Basel, Switzerland), including Cobas^®^ MTB-RIF/INH, and the BD MAX MDR-TB (BD, Franklin Lakes, NJ, USA) assays. Both can identify RR-TB and INHR; hence, downstream national data review would be valuable to repeat as described here.

It is essential that we limit the development and spread of DR-TB to reach the goal of eliminating TB. Our results highlight the importance of good follow-up post-treatment, including potential repeat screening with a culture-based test within 6 months of treatment conclusion and use of drug resistance testing, particularly among males and PLHIV due to their higher recurrence risk. Individuals with INHR-TB are also at higher risk of developing RR-TB upon recurrence, showing the importance of baseline INHR testing.
